# Knowledge, Attitude, and Practices of Complementary and Alternative Medication Usage in Patients of Type II Diabetes Mellitus

**DOI:** 10.7759/cureus.5357

**Published:** 2019-08-09

**Authors:** Ravi Raja, Vikash Kumar, Muhammad Ali Khan, Khalid A Sayeed, Syed Zohaib Maroof Hussain, Amber Rizwan

**Affiliations:** 1 Internal Medicine, New Medical Center, Al Ain, ARE; 2 Internal Medicine, Jinnah Sindh Medical University, Karachi, PAK; 3 Otolaryngology, Dow University of Health Sciences, Karachi, PAK; 4 Medicine, Liaquat College of Medicine and Dentistry, Darul Sehat Hospital, Karachi, PAK; 5 Surgery, Aga Khan University Hospital, Karachi, PAK; 6 Family Medicine, Dr. Ruth Pfau Hospital, Karachi, PAK

**Keywords:** knowledge attitude practice, alternative and complementary medicine, complementary medicine, diabetes mellitus, cupping therapy, herbal treatment

## Abstract

Introduction

Complementary and alternative medicine (CAM) is becoming popular among individuals affected by chronic diseases, such as diabetes mellitus. We aimed to determine the knowledge, attitude, and practices of complementary and alternative medicine use among type 2 diabetes patients in Karachi, Pakistan.

Methods

An observational, prospective, cross-sectional study was conducted in the institute of diabetology in a tertiary care hospital in Pakistan from 1st March 2018 till 31st August 2018. All patients of type 2 diabetes mellitus attending the clinic for routine follow-up visits during the study period were interviewed. Their demographic characteristics, clinical data, and knowledge, attitude, practices towards use of CAM products were assessed. Data was managed using SPSS for Windows version 16.0 (SPSS Inc, Chicago, IL).

Results

CAM therapies were being used by 151 (57.8%) individuals. Herbs (n = 121; 80.1%), specific diets (n = 98; 64.9%), and cupping (n = 68; 45.0%) were the most readily utilized CAM practices. CAM practices were associated with diabetes-related complications [p < 0.000; Odds Ratio (OR) 2.57; Confidence Interval (CI) 1.53, 4.34], poor glycemic control (p < 0.000; OR 0.29; CI 0.17, 0.5), lack of trust in pharmaceutical products (p < 0.000; OR 5.08; CI 2.28, 11.32), poor patient-doctor relationship (p = 0.06; OR 1.47; CI 0.26, 8.17), CAM products being readily available and cheaper (p < 0.000; OR 6.1; CI 3.02, 12.32), and belief that CAM products have fewer side effects (p < 0.000; OR 12.32; CI 6.83, 22.22) and can help in diabetes control (p < 0.000; OR 35.76; CI 16.79, 76.15).

Conclusion

Use of complementary medicine products among Pakistani diabetic population is high. Herbs and specific diets were common modes of CAM practices. Use of CAM showed significant association with female gender, older age, unemployment, longer duration of diabetes, diabetes-related complications, and poor glycemic control.

## Introduction

Diabetes mellitus (DM) is a chronic disorder which cannot be cured, but is managed with modifying lifestyle to maintain blood sugar levels within the normal range. The conventional method of managing type 2 DM (T2DM) involves oral glucose lowering drugs and/or insulin therapy. Despite decades of research and development of new classes of anti-diabetic drugs, managing diabetes effectively remains a grave challenge for clinicians. Therapeutic compliance, medication access, counseling, regular monitoring of blood sugar levels, access to health care services, and quality of management are some factors governing the efficacy of diabetes treatment [1].

However, not all patients are benefitted by conventional anti-diabetic therapy and some patients of T2DM were supplemented with complementary alternative medicines (CAM) in addition to conventional medicines [2]. Wanchai and Phrompayak made two categories of CAM therapy - natural products and practices of mind and body. Herbs, minerals and vitamins, essential oils, and probiotics are included in natural products. Practices of mind and body vary according to individual needs and include yoga, chiropractic manipulation, meditation, massage, acupuncture, cupping, and many more [2]. In a study with patients of chronic diseases, 64% were found to be using CAM. Out of these, 35.5% were using CAM for diabetes mellitus. They reported vitamins (48.2%) and herbal medicines (26.4%) as commonly used CAM. Higher education status, higher income, and aged above 50 years were independently associated with CAM use. Many patients (77.6%) reported improved condition with use of CAM [3].

Although patients might perceive natural products of CAM to be safer and more effective as compared to pharmaceutical drugs and may use them to alleviate symptoms of chronic diseases, some have deduced that CAM may worsen health. In a study with diabetic patients, CAM users were associated with poor glycemic control and higher disease severity [4]. In another study with 29% diabetics using CAM for diabetes specifically, CAM users had poor cardiometabolic control with elevated low density lipoproteins and poor medication adherence [5]. The aim of this study was to evaluate knowledge, attitude, practices (KAP) of diabetic patients towards use of CAM products.

## Materials and methods

It was an observational, prospective, cross-sectional study conducted in the institute of diabetology in a tertiary care hospital in Pakistan. Data was collected from 1st March 2018 till 31st August 2018. Non-randomized, convenient, consecutive sampling technique was utilized. Informed verbal consent was obtained from all participants and ethical approval institutional review board was obtained.

All patients of type 2 diabetes mellitus attending the clinic for routine follow-up visits during the study period were interviewed. Their demographic characteristics including gender, age, marital status, education status, employment status, and smoking status were recorded. Clinical characteristics included in the study were duration of T2DM, mode of treatment of T2DM (oral medications and/or insulin), complications associated with T2DM, glycemic control, and other medical co-morbidities. In order to assess KAP of the participants towards use of CAM products, questionnaire was adapted from Al-Eidi et al. [6]. There were three questions regarding knowledge, four regarding attitude, and seven reasons of the attitude. For participants who practiced CAM use, type of CAM, recommender of CAM, and user behaviour was assessed [6].

Data was managed using SPSS for Windows version 16.0 (SPSS Inc, Chicago, IL). For continuous variables, mean and standard deviation was calculated. For categorical variables, frequency and percentages were calculated. Demographic data, clinical data, and KAP characteristics were correlated with the use of CAM using Pearson’s Chi-square test. P value ≤0.05 was taken as significant. Odds ratio (OR) along with confidence interval (CI) was considered as determinants of strength.

## Results

Two hundred and ninety-three participants were approached for the study. Fifteen participants refused and 17 responses were omitted due to incomplete information. Remaining 261 responses were included in the analysis. There were 159 (60.9%) men and 102 (39.1%) women. Their mean age was 61 ± 13 years (range: 54-78 years). Most of the participants were of age more than 60 years. More than half of the sample was married (64.7%). About 14.5% participants were uneducated and 41% were employed. All patients were diagnosed with T2DM; 8% were newly diagnosed. The mean duration of diagnosis was 8.6 ± 3.7 years (range: 0-17 years). About half of the patients (49%) were taking oral anti-diabetic medications and more than 35% had developed diabetes-related complications. The most common complication was peripheral neuropathy (43%). Adequate glycemic control was present in 34%. Other medical conditions were present in 185 (70.9%) participants. All demographic and clinical characteristics of the patients were summarized in Table 1.

**Table 1:** Frequency of socio-demographic and clinical characteristics of the participants (N = 261) HbA1c: Glycosylated haemoglobin type A1c; T2DM: Type 2 diabetes mellitus.

Sociodemographic characteristics	Frequency n (%)	Clinical characteristics	Frequency n (%)
Gender		Duration of T2DM diagnosis	
Male	159 (60.9%)	Newly diagnosed	21 (8.1%)
Female	102 (39.1%)	<5 years	87 (33.3%)
Age		5 to <10 years	101 (38.7%)
≤40 years	52 (19.9%)	Greater than 10 years	52 (19.9%)
41-59 years	96 (36.7%)	Medications used for T2DM	
≥60 years	113 (43.3%)	Oral hypoglycemic drugs	128 (49.1%)
Marital status		Insulin	82 (31.4%)
Married	169 (64.7%)	Both	51 (19.5%)
Never married	28 (10.7%)	T2DM-related complications	
Divorced/Widowed	64 (24.5%)	No	170 (65.1%)
Education		Yes	91 (34.8%)
Illiterate	38 (14.5%)	T2DM complications (n = 91)	
Primary / secondary / high school	128 (49.1%)	Peripheral neuropathy	29 (43.3%)
Bachelors and above	95 (36.3%)	Cardiovascular disease	19 (28.3%)
Employment status		Diabetic foot wound/ulcer	18 (26.9%)
Employed	107 (41.0%)	Retinopathy	16 (23.8%)
Unemployed	62 (23.7%)	Nephropathy	8 (11.9%)
Retired	92 (35.2%)	Others	1 (1.4%)
Smoking status		Glycemic control	
Smoker	104 (39.8%)	HbA1c ≤ 7%	89 (34.1%)
Non-smoker	58 (22.2%)	HbA1c > 7%	172 (65.9%)
Ex-smoker	99 (37.9%)	Medical Co morbidity status	
		Hypertension	122 (46.7%)
		Hyperlipidemia	111 (42.5%)
		Ischemic heart disease	109 (41.7%)
		Obesity	98 (37.5%)
		Others	12 (4.6%)

In Table 2, the knowledge, attitude and reasons of attitude of the study participants towards the use of complementary medications for T2DM are summarized. Almost all of the patients (n = 260; 99.6%) had heard of CAM and half of the sample (n = 112; 42.9%) knew that CAM can be effective and 101 (38.7%) believed that CAM were safe for them. When the attitude of participants was assessed towards use of CAM, it was deduced that 77% would first discuss using CAM with their current physician, 8% would use CAM even if their physicians did not advise them to, and 49% will also use their T2DM medications along with CAM. Majority of the patients (57.5%) mentioned that they will use CAM because of lesser side effects and 49% believed that CAM will help control their blood sugar levels. Delayed doctor appointment was an important reason in 35% participants, cost was a factor in 28%, poor physician-patient relationship in 4%, lack of trust in modern medicines in 19.5%, and in 2.3% cases the doctors recommended using CAM to the patients. All responses of knowledge, attitude, and reasons of attitude are summarized in Table 2.

**Table 2:** Knowledge, attitude, and reasons of attitude of the participants regarding complementary alternative medications (N = 261) CAM: Complementary alternative medications; T2DM: Type 2 diabetes mellitus.

Variables	Frequency n (%)
Knowledge	
Have you heard of CAM?	260 (99.6%)
Do you believe that CAM products are effective?	112 (42.9%)
Do you believe that CAM products are safe?	101 (38.7%)
Attitude	
If you want to use CAM, will you discuss with the physician?	201 (77.1%)
If you want to use CAM, will you combine it with T2DM medications?	129 (49.4%)
Will you advise a family member with T2DM to use CAM?	82 (31.4%)
If your physician instructs you not to use CAM, would you follow him?	21 (8.1%)
Reasons of attitude	
Believe CAM have fewer side effects	150 (57.5%)
Believe CAM can help the diabetes control	128 (49.1%)
Long appointment intervals to see physician	92 (35.2%)
CAM are easily available and cheaper	72 (27.6%)
Lack of trust in pharmaceutical drugs	51 (19.5%)
Poor physician-patient communication	10 (3.8%)
Doctor suggested using CAM	6 (2.3%)
Using CAM for T2DM	
Yes	151 (57.8%)
No	90 (34.5%)

As seen in Table 2, 151 (57.8%) participants claimed that they use alternative medicines. Out of these, 82 [82/151 (54.3%); 82/261 (31.4%)] patients claimed that they were using CAM for their diabetes. The practices of these patients are summarized in Table 3 which shows that herbs (n = 121; 80.1%) were the most common form of CAM used. Other family members (n = 44; 29.1%) and traditional healers (n = 38; 25.2%) commonly recommended CAM. In two (1.3%), the physicians had prescribed CAM. There were only 41/151 (27%) patients who were combining their CAM with T2DM medications, rest were using CAM alone, 24% claimed that they will continue to use CAM, 19% were satisfied with their products, and 14.5% had discussed using CAM with their physicians. The characteristics of CAM users are summarized in Table 3.

**Table 3:** Responses of participants who use complementary alternative medications (N = 151) Responses may be more or less than 151 due to multiple preferences. CAM: Complementary alternative medications; T2DM: Type 2 diabetes mellitus

Variables	Frequency n (%)
CAM Practices used	
Herbs	121 (80.1%)
Treatments based in a specific diet	98 (64.9%)
Cupping	68 (45.0%)
Nutritional supplements (vitamins and minerals)	28 (18.5%)
Spiritual healing	26 (17.2%)
Honeybee products	12 (7.9%)
Medical massage	10 (6.6%)
Recommendation of CAM given by	
Family	44 (29.1%)
Traditional healer	38 (25.2%)
Friend	24 (15.9%)
Pharmacist	20 (13.2%)
Herbalist	19 (12.6%)
Dietician	4 (2.6%)
Physician	2 (1.3%)
User behaviour	
Have you ever used CAM for a condition other than T2DM?	101 (66.8%)
Have you ever used CAM for T2DM?	82 (54.3%)
Do you combine CAM and T2DM medication?	41 (27.2%)
Will you use CAM product again?	36 (23.8%)
Are you satisfied with the CAM products?	29 (19.2%)
Do you discuss with physician about CAM products?	22 (14.5%)

Demographic characteristics, knowledge, attitude, and practices of the participants were correlated with use of CAM. Demographic and clinical characteristics which showed significant association to the use of CAM were gender (p < 0.000; OR 0.24; CI 0.14, 0.42), age (p < 0.000), marital status (p = 0.04), education status (p < 0.000), employment status (p < 0.000), duration of diabetes (p < 0.000), diabetes-related complications (p < 0.000; OR 2.57; CI 1.53, 4.34), and glycemic control (p < 0.000; OR 0.29; CI 0.17, 0.5). KAP-related characteristics which were significantly correlated with the use of CAM included lack of trust in pharmaceutical products (p < 0.000; OR 5.08; CI 2.28, 11.32), longer time intervals to see physicians (p = 0.001; OR 0.43; CI 0.26, 0.72), poor patient-doctor relationship (p = 0.06; OR 1.47; CI 0.26, 8.17), CAM products being readily available and cheaper (p < 0.000; OR 6.1; CI 3.02, 12.32), and belief that CAM products have fewer side effects (p < 0.000; OR 12.32; CI 6.83, 22.22) and can help in diabetes control (p < 0.000; OR 35.76; CI 16.79, 76.15) (Table 4).

**Table 4:** Relationship of participants characteristics with usage of complementary alternate medicines (N = 261) CAM: Complementary alternative medications; CI: Confidence interval; T2DM: Type 2 diabetes mellitus.

Variables	Frequency n (%)	Use of CAM		P value	Odds Ratio	CI (95%)
		Yes (n = 151; 57.8%)	No (n = 110; 42.1%)			
Gender						
Male	159 (60.9%)	72 (45.3%)	87 (54.7%)	<0.000	0.24	0.14, 0.42
Female	102 (39.1%)	79 (77.4%)	23 (22.5%)			
Age in years						
≤40 years	52 (19.9%)	18 (34.6%)	34 (65.3%)	<0.000	NA	NA
41-59 years	96 (36.7%)	49 (51.0%)	47 (49.0%)			
≥60 years	113 (43.3%)	84 (74.3%)	29 (25.7%)			
Marital status						
Married	169 (64.7%)	97 (57.4%)	72 (42.6%)	0.04	NA	NA
Never married	28 (10.7%)	11 (39.3%)	17 (60.7%)			
Divorced / Widowed	64 (24.5%)	43 (67.2%)	21 (32.8%)			
Education						
Illiterate	38 (14.5%)	29 (76.3%)	9 (23.7%)	<0.000	NA	NA
Primary / secondary / high school	128 (49.1%)	95 (74.2%)	33 (25.8%)			
Bachelors and above	95 (36.3%)	27 (28.4%)	68 (71.6%)			
Employment status						
Employed	107 (41.0%)	35 (32.7%)	72 (67.2%)	<0.000	NA	NA
Unemployed	62 (23.7%)	48 (77.4%)	14 (22.6%)			
Retired	92 (35.2%)	68 (73.9%)	24 (26.1%)			
Duration of T2DM diagnosis						
Newly diagnosed	21 (8.1%)	7 (33.3%)	14 (66.7%)	<0.000	NA	NA
<5 years	87 (33.3%)	37 (42.5%)	50 (57.5%)			
5 to <10 years	101 (38.7%)	68 (67.3%)	33 (32.7%)			
Greater than 10 years	52 (19.9%)	39 (75.0%)	13 (25.0%)			
Medications used for T2DM						
Oral hypoglycemic drugs	128 (49.1%)	68 (53.1%)	60 (46.9%)	0.25	NA	NA
Insulin	82 (31.4%)	53 (64.6%)	29 (35.4%)			
Both	51 (19.5%)	30 (58.8%)	21 (41.2%)			
T2DM-related complications						
No	170 (65.1%)	112 (65.9%)	58 (34.1%)	<0.000	2.57	1.53, 4.34
Yes	91 (34.8%)	39 (42.9%)	52 (57.1%)			
Glycemic control						
HbA1c ≤ 7%	89 (34.1%)	34 (38.2%)	55 (61.8%)	<0.000	0.29	0.17, 0.5
HbA1c > 7%	172 (65.9%)	117 (68.1%)	55 (31.9%)			
Medical comorbidities						
Yes	185 (70.9%)	103 (55.7%)	82 (44.3%)	0.26	0.73	0.42, 1.27
No	76 (26.1%)	48 (63.2%)	28 (36.9%)			
Lack of trust in pharmaceutical drugs						
Yes	51 (19.5%)	43 (84.3%)	8 (15.7%)	<0.000	5.08	2.28, 11.32
No	210 (80.5%)	108 (51.4%)	102 (48.6%)			
Waiting time intervals to see physicians						
Yes	92 (35.2%)	41 (44.6%)	51 (55.4%)	0.001	0.43	0.26, 0.72
No	169 (64.8%)	110 (65.1%)	59 (34.9%)			
Poor physician-patient communication						
Yes	10 (3.8%)	3 (%)	7 (%)	0.06	0.3	0.08, 1.18
No	251 (96.2%)	148 (%)	103 (%)			
Doctor suggesting it						
Yes	6 (2.3%)	4 (%)	2 (%)	0.65	1.47	0.26, 8.17
No	255 (97.7%)	147 (%)	108 (%)			
Availability and better value for money						
Yes	72 (27.6%)	61 (%)	11 (%)	<0.000	6.1	3.02, 12.32
No	189 (72.4%)	90 (%)	99 (%)			
Belief in that CAM has fewer side effects						
Yes	150 (57.5%)	122 (%)	28 (%)	<0.000	12.32	6.83, 22.22
No	111 (42.5%)	29 (%)	82 (%)			
Belief in that CAM can help diabetes control						
Yes	128 (49.1%)	118 (%)	10 (%)	<0.000	35.76	16.79, 76.15
No	133 (50.9%)	33 (%)	100 (%)			

## Discussion

Almost one-third of all diabetic patients were found to be using CAM for their diabetes. Herbs and specific diets were common modes of CAM practices. Family members were the common recommenders of CAM. Use of CAM showed significant association with female gender, older age, divorced/widow marital status, lower education, unemployment, longer duration of diabetes, diabetes-related complications and poor glycemic control. KAP-related characteristics which were significantly correlated with the use of CAM included lack of trust in pharmaceutical products, longer time intervals to see physicians, poor patient-doctor relationship, CAM products being readily available and cheaper, and belief that CAM products have fewer side effects and can help in diabetes control.

To the best of our knowledge, this is the first study to provide detailed report of KAP of Pakistani diabetic individuals regarding the use of CAM. Kamran et al. have recently published a significant report regarding the pattern of CAM practices and CAM-related beliefs among diabetic patients in Pakistan [7]. However, this study was conducted in one centre only and its results cannot be generalized. Furthermore, it included all patient characteristics on one instance only and did not follow the patients over time to study the actual effects of CAM on their health. Other causes of poor glycemic control and diabetes-related complications cannot be eliminated.

In a qualitative analysis from Pakistan, religious practices (37.5%), herbal (15.5%), and household remedies (9.4%) were the main CAM practices among diabetic patients. Patients reported that their practices had significant positive impacts on their health. The main motivator of CAM use was the desire to find cure of diabetes. This desire was strongly triggered by pressure from the family and community [8]. In another recent analysis from Pakistan, 41% patients supported combination of CAM and conventional therapy for T2DM and only 3% supported CAM alone. A slight majority (55%) trusted CAM for T2DM, 47% favored CAM, and 49% observed no change in their diabetes with CAM. Prayers, herbs (bitter gourd), and multivitamins were the commonly used modes of CAM [7]. In another local analysis, more than half (53%) diabetics were using CAM. Less education and concomitant kidney disease were important predictors of CAM use [9].

In a KAP study from Saudi Arab, almost all patients knew about CAM, 27% found them safe, and 26% found them effective. A high majority (90%) reported that they will first discuss CAM usage with their physicians and 44% preferred combining CAM with their conventional therapy. Herbs were the most common CAM (31%), followed by wet cupping (20.5%) and nutritional supplements (18%) [6]. In another Saudi meta-analysis, the overall frequency of CAM use in diabetic patients was 32% with herbs including fenugreek and black seeds being the most common [10]. Predictors of CAM use were age above 51 years, unemployment, and the participants’ knowledge about the effectiveness of CAM products [6]. Herbs and specific diets were also the common mode of CAM in this study. However, in a study with Thai diabetics, Tai chi (a form of Chinese martial arts), prayer, and meditation were the common practices. They reported female gender, age 40-69 years, and diabetes duration less than 10 years as significant correlators of CAM use [2]. Female gender and older age was also correlated with CAM use in our study, but results were contradictory as far as duration of diabetes is concerned.

In a Nigerian study, 67% diabetics were using herbal products for glycemic control. Out of these, only 32% had disclosed it to their physicians. The common reasons included safety (74%) and affordability (60%). They reported older age, less education, longer duration of diabetes, diabetes management using oral hypoglycaemics, family history of diabetes, and neuropathy as predictors of herbal medicine use [1]. In another Nigerian study, use of CAM resulted in better glycemic control and an improved lipid profile [11]. The results are contrary to our study where users of CAM depicted worse glycemic control. Poor glycemic and cardiometabolic control with CAM has been established in other studies too [4,5]. In an Iranian study, glycemic control and foot care had the strongest positive correlations with religious practices [12]. Wet and dry cupping therapies also known as Hijama are also becoming popular in alternative treatment of diabetes [13]. Cupping therapy in diabetic patients resulted in significant reduction in their hemoglobin A1C, pre-prandial and post-prandial blood sugar levels, serum triglyceride, serum cholesterol, low density lipoprotein and espartos transferees. There was a significant increase in high density lipoproteins [14].

Use of complementary and alternative medicines for chronic illnesses including diabetes mellitus is becoming more popular day by day. With evolving patient preferences, clinicians as well as researchers have to shift their focus to this mode of treatment. Clinicians should encourage healthy discussions with the patients to comprehend their views regarding CAM. Clinicians and researchers should join hands to initiate safety and efficacy trials on common herbal products used for diabetes. Presence of concrete evidence in the form of randomized controlled trials will help both patients and clinicians regarding use of a certain complementary medicine product.

## Conclusions

Use of complementary medicine products among Pakistani diabetic population is high. Herbs and specific diets were common modes of CAM practices. Use of CAM showed significant association with female gender, older age, lower education, unemployment, longer duration of diabetes, diabetes-related complications, and poor glycemic control. KAP-related characteristics which were significantly correlated with the use of CAM included lack of trust in pharmaceutical products, longer time intervals to see physicians, poor patient-doctor relationship, CAM products being readily available and cheaper, and belief that CAM products have fewer side effects and can help in diabetes control.
